# Stochastic models of cellular circadian rhythms in plants help to understand the impact of noise on robustness and clock structure

**DOI:** 10.3389/fpls.2014.00564

**Published:** 2014-10-21

**Authors:** Maria L. Guerriero, Ozgur E. Akman, Gerben van Ooijen

**Affiliations:** ^1^Systems Biology Ireland, University College DublinDublin, Ireland; ^2^Centre for Systems, Dynamics and Control, College of Engineering, Mathematics and Physical Sciences, University of ExeterExeter, UK; ^3^Institute of Molecular Plant Sciences, University of EdinburghEdinburgh, UK

**Keywords:** circadian clocks, plants, *Arabidopsis thaliana*, *Ostreococcus tauri*, modeling, stochasticity, intrinsic noise, extrinsic noise

## Abstract

Rhythmic behavior is essential for plants; for example, daily (circadian) rhythms control photosynthesis and seasonal rhythms regulate their life cycle. The core of the *circadian clock* is a genetic network that coordinates the expression of specific clock genes in a circadian rhythm reflecting the 24-h day/night cycle. Circadian clocks exhibit stochastic noise due to the low copy numbers of clock genes and the consequent cell-to-cell variation: this *intrinsic* noise plays a major role in circadian clocks by inducing more robust oscillatory behavior. Another source of noise is the environment, which causes variation in temperature and light intensity: this *extrinsic* noise is part of the requirement for the structural complexity of clock networks. Advances in experimental techniques now permit single-cell measurements and the development of single-cell models. Here we present some modeling studies showing the importance of considering both types of noise in understanding how plants adapt to regular and irregular light variations. Stochastic models have proven useful for understanding the effect of regular variations. By contrast, the impact of irregular variations and the interaction of different noise sources are less well studied.

## MATHEMATICAL MODELS OF THE PLANT CIRCADIAN CLOCK

The rotation of planet Earth around its axis generates predictable daily oscillations in sunlight. Plants require sunlight to derive energy via photosynthesis, and have therefore evolved intricate molecular clocks to link growth and cellular metabolism to the appropriate phase of the solar cycle (Harmer, 2009). Creating an internal cellular rhythm to match the rhythmic external environment confers a major fitness benefit to the plant (Dodd et al., 2005; Graf et al., 2010). The circadian clock regulatory network generates oscillations in gene expression and is able to adapt to environmental conditions by synchronizing (or *entraining*) to light/dark (LD) cycles (Dunlap et al., 2003). Such oscillations persist under conditions of constant light (LL) or constant darkness. Hallmark features of the circadian clock are the ability to function both with and without entrainment, robustness against perturbations, and the flexibility to adapt to environmental changes resulting from weather and seasons (Akman et al., 2008, 2010b; Troein et al., 2009; Edwards et al., 2010; Gould et al., 2013).

The most studied plant circadian clock is that of the model species *Arabidopsis thaliana* (Harmer, 2009). The heart of the *Arabidopsis* transcriptional clock network is a double negative feedback loop between the morning-phased heterodimeric transcription factor complex of LHY/CCA1 and the evening-expressed pseudoresponse regulator TOC1. Additional feedback loops exist between LHY/CCA1 and PRR7/9, and LHY/CCA1 and the Evening Complex (Nusinow et al., 2011; Huang et al., 2012). Several mathematical models of the *Arabidopsis* clock network have been developed, starting from a minimal model comprising one negative–positive feedback loop (Locke et al., 2005), through models of intermediate complexity which include multiple loops and light inputs (Locke et al., 2006; Pokhilko et al., 2010), to the most recent models that incorporate more detailed biochemical mechanisms such as post-translational modification (Pokhilko et al., 2012). Most published models of the circadian clock in *Arabidopsis* are continuous deterministic models based on ordinary differential equations (ODEs). An alternative approach adopts linear time invariant (LTI) models (Dalchau et al., 2010; Herrero et al., 2012). These models, which have the advantage of being computationally more tractable than kinetic ODE models, have been used to investigate how the *Arabidopsis* clock combines timing information from the central rhythm generator and light-signaling pathways to control output rhythms such as cytosolic calcium oscillations (Dalchau et al., 2010), and to infer regulatory interactions between clock components, thereby yielding new predicted clock architectures (Herrero et al., 2012). However, LTI models fail to represent the system’s behavior when nonlinearity is fundamental: in particular, they cannot simulate sustained oscillations in constant conditions. For a comprehensive review on *Arabidopsis* clock modeling see (Bujdoso and Davis, 2013).

Ordinary differential equation models provide a good representation of the dynamical behavior of a population of cells, or a whole organism, but represent the dynamics of a single-cell less accurately. This is due to the underlying assumption that discrete variables representing molecule copy numbers can be approximated by continuous ones representing concentrations when the number of molecules is large. This assumption is generally valid in a cell population but not in single-cells, particularly in gene regulatory networks where genes can be present in low copy numbers.

The discrete stochastic modeling paradigm differs from the ODE approach in two main ways: firstly, model variables represent discrete molecule numbers rather than continuous concentrations, and secondly, the time evolution of the model is obtained by taking into account the probability of each reaction to occur, computed assuming the mass action law or other kinetics (Michaelis-Menten, Hill, etc.). Stochastic models are generally simulated using a stochastic simulation algorithm (SSA; Gillespie, 1977; Gibson and Bruck, 2000; Cao et al., 2007). The time course of a model variable obtained from a single stochastic simulation is considered to represent the dynamic behavior of a molecular species in a single-cell, while the average of multiple stochastic simulation time courses is taken to represent its behavior in a cell population.

Until recently, experimental techniques were focused on measuring large populations of cells, meaning that single-cell computational results did not have an experimental counterpart. Recent advances in experimental techniques and high-resolution imaging have allowed for measurements of smaller cell populations. Indeed, even single-cell measurements are becoming feasible (Wang and Bodovitz, 2010; Yang et al., 2010; Lee et al., 2011; Enoki et al., 2012), generating an interesting avenue for single-cell stochastic modeling to assist future studies into cell-autonomous timekeeping mechanisms. In this article, we will address recent advances and future perspectives on stochastic plant circadian clock modeling.

## INTRINSIC NOISE IN THE PLANT CIRCADIAN CLOCK

The importance of stochasticity in genetic networks is well known (McAdams and Arkin, 1999), and arises from the fact that the molecules involved are generally present in very low concentrations. This kind of stochasticity, referred to as *intrinsic* noise, has been observed in circadian clock networks of animal, plant, and fungal species, and has been shown to increase the robustness of oscillations in the concentrations of network components (Gonze et al., 2002a,b; Forger and Peskin, 2005; Akman et al., 2009, 2010a).

The role of intrinsic noise in the *Arabidopsis* circadian clock was previously investigated using a stochastic model (Guerriero et al., 2012). A key finding was that the fluctuations in protein and gene expression induced by intrinsic noise caused desynchronization between single-cell oscillations (i.e., in individual stochastic simulations) under LL conditions. This desynchronization, and resulting phase diffusion, caused damping of the oscillations simulated at the cell population level by averaging multiple stochastic simulations. The predicted damping of circadian rhythms was consistent with the experimentally observed oscillations in LL. Additionally, moderate intrinsic noise was shown to accelerate the re-entrainment of the plant clock to experimentally imposed sudden changes to the LD cycle. These results indicate that a certain level of intrinsic noise may be essential for the *Arabidopsis* clock to properly adapt to a noisy environment, implying a need for average clock molecule expression levels to be tuned appropriately.

**FIGURE 1:**
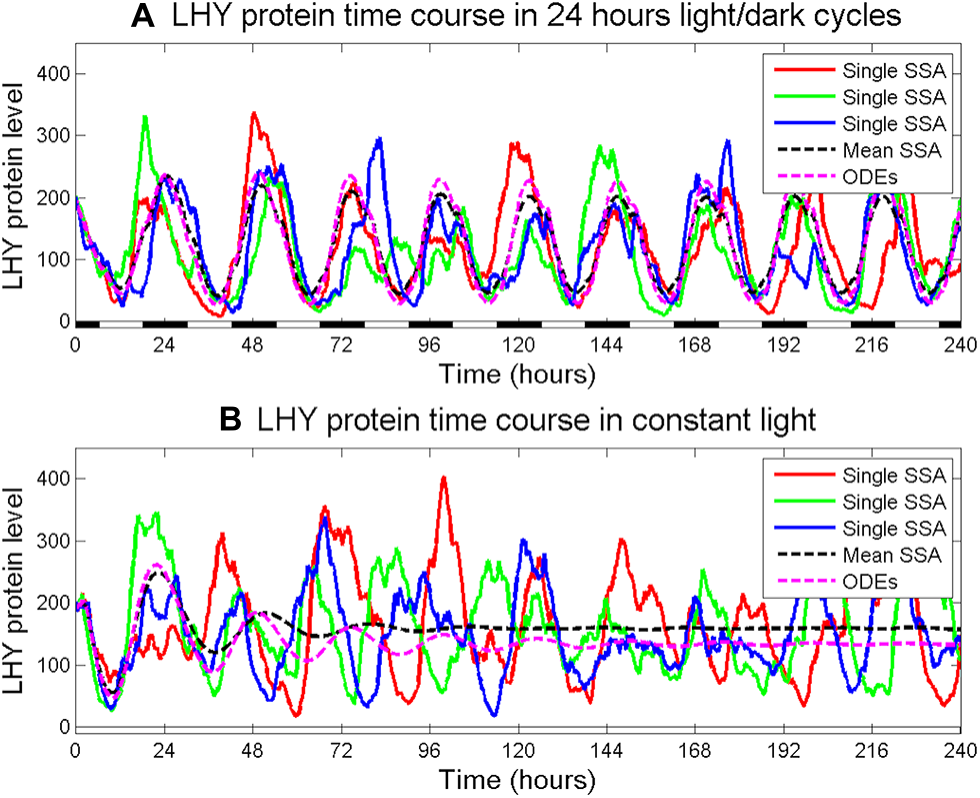
**Effect of intrinsic noise on oscillation robustness in *Ostreococcus tauri* in entrained and constant conditions. (A)** 12 h:12 h light/dark (LD) cycles. **(B)** Constant light (LL). For each light condition, three independent realizations of the system’s dynamics obtained using the stochastic simulation algorithm (SSA) are shown, together with the deterministic behavior (dotted pink lines) and the mean stochastic behavior obtained by averaging over 10000 independent runs (dotted black lines). Note how the greater variability between the independent runs observed in LL compared to LD causes the mean population behavior to exhibit damped oscillations in LL **(B)** and sustained oscillations in LD **(A)**. Interestingly, some individual cells can drift out of phase even in LD; however, the light entrainment is able to limit these occurrences so that, as a population, the cells oscillate regularly.

An additional and useful model organism in this context is the alga *Ostreococcus tauri,* as it is naturally unicellular and contains a plant-like circadian clock that includes a feedback loop between TOC1 and a single ortholog of *Arabidopsis* CCA1 and LHY, named CCA1 (Corellou et al., 2009). *Ostreococcus* offers a cellular model system of minimal genomic complexity, with a genome roughly the size of yeast (Derelle et al., 2006), as well as minimal cellular complexity, with only one chloroplast and mitochondrion per cell (Henderson et al., 2007). Many of the components of additional loops found in the transcriptional oscillator of higher plants are absent, yet *Ostreococcus* cells exhibit all the cellular circadian behaviors observed in higher plants, both in free-running and entrained conditions (Corellou et al., 2009). Both an ODE model (Troein et al., 2011) and a stochastic model (Akman et al., 2010a) have been developed for this reduced plant clock. Comparing these models yields identical predictions for oscillations of large cell populations (Akman et al., 2010a). By contrast, when simulating rhythms in single cells assuming a daily average of 50 molecules per cell, the high intrinsic noise yields significant departures from the corresponding deterministic formulation. In particular, single stochastic simulations show persistent oscillations both under entrainment and in LL (**Figure 1**). However, without entrainment, the oscillations are not synchronized across different cells, causing the mean behavior of the system to dampen. This result suggests that when copy numbers of clock components are low, stochasticity promotes oscillations, and helps cells to keep time autonomously.

Low molecule numbers per cell have been experimentally verified in *Ostreococcus* (van Ooijen et al., 2011). Throughout the day, TOC1 cycled between 10 and 150 molecules per cell and CCA1 between 80 and 400 molecules per cell. Additionally, degradation rates of both clock proteins were observed to fluctuate over a 24-h period. TOC1 degradation rates were phase-dependent in response to LD transitions and depended on photoperiods (i.e., seasons). By contrast, CCA1 degradation rates were truly circadian and rhythmic at the same phase, regardless of the entrainment conditions. Given that properly phased protein degradation appears to be even more important to timekeeping (van Ooijen et al., 2011) than rhythmic protein synthesis (O’Neill et al., 2011), it might prove revealing to incorporate degradation rate rhythms into future stochastic models.

Phase-dependent changes in degradation rates of *Arabidopsis* LHY and TOC1 have been observed in cell extracts (Mas et al., 2003; Song and Carré, 2005), but accurately tracing degradation *in vivo* to establish the peak phase of clock protein degradation is much more challenging in *Arabidopsis* than in *Ostreococcus*. Similarly, clock protein molecule numbers in *Arabidopsis* are hard to measure directly, but have been estimated from gene expression measurements to be of the order of a few 100 proteins per cell (Guerriero et al., 2012; Supplementary data). Comparing the wealth of available experimental luminescence results to stochastic simulations generated by models with different system sizes (i.e., different average molecule counts) confirmed this estimate computationally. These results mean that, in principle, clock components are sufficiently lowly expressed for intrinsic noise to exist at significant levels in the higher plant clock. It certainly would be a valuable exercise to ascertain experimentally, as well as theoretically, whether the key role of intrinsic noise for cell-autonomous rhythms observed in *Ostreococcus* translates over to the more elaborately regulated higher plant system. However, an important difference between unicellular organisms such as *Ostreococcus* and higher plants such as *Arabidopsis* is the impact of intercellular interactions and spatial structure, which potentially introduce an extra level of complexity when studying the plant circadian clock. In animals, the master clock in the suprachiasmatic nucleus (SCN) is characterized by strong intercellular coupling, yielding a timing mechanism that is robust to environmental noise, whilst weaker coupling is observed in the peripheral clocks, enabling them to respond flexibly to signals from the SCN and other systems (e.g., hormonal and metabolic signals; Abraham et al., 2010). Plants appear to have a different architecture, in which a heterogeneous network of weakly coupled oscillators achieves accurate timing through strong coupling to the external light/dark cycle in leaves (Wenden et al., 2012). By analogy to the mammalian system, it has been proposed that the clock in *Arabidopsis* roots is a peripheral clock slaved to a photosynthesis-generated metabolic signal from a master oscillator in green tissues (James et al., 2008). In a recent breakthrough paper, photosynthetically derived sugars were proven to provide key metabolic input to the circadian oscillator in a process involving the clock protein PRR7 (Haydon et al., 2013), and it is likely that this signal could contribute to the orchestration of rhythmic transcripts in photosynthetically inactive parts of the plant.

## EXTRINSIC NOISE IN THE PLANT CIRCADIAN CLOCK

**FIGURE 2:**
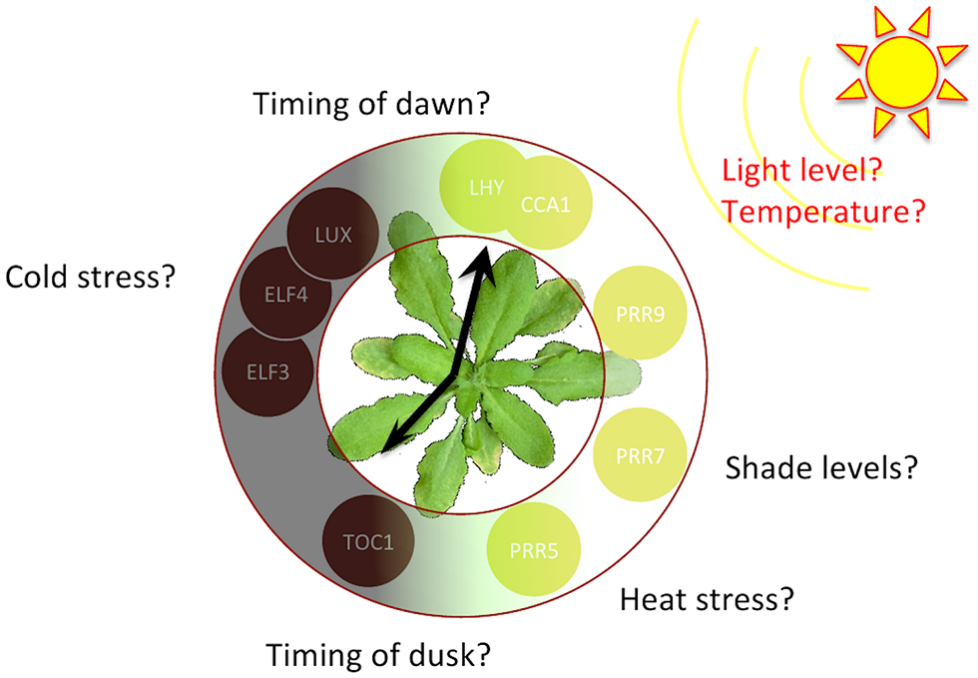
**Some of the sources of extrinsic noise particularly relevant to plants.** Primary physical variations depending on weather and seasons are in red font, and translated physiologically relevant factors for a plant system are in black. Peak expression phase of the *Arabidopsis* clock components are provided on a 24-h circular clock diagram.

In addition to intrinsic molecular noise, circadian clocks are also subject to *extrinsic* noise; weather and seasons generate fluctuations in environmental factors like photoperiod, light intensity, and temperature. This type of noise is particularly relevant to plants, which have to be more robust to environmental changes compared to animals due to their immobility and lack of temperature regulation (**Figure 2**). The obvious need for sunlight in photosynthesis directly links environmental conditions to cellular metabolism and survival; it is therefore of great importance to plants to accurately trace the timing of dusk and dawn throughout the seasons. This timekeeping allows anticipation of predictable daily changes, and proper alignment of cellular metabolism to the most efficient phase of the day. Weather is intrinsically unpredictable, however, and therefore clocks have to be buffered against fluctuations in light level and temperature. Models have been increasingly used to understand the molecular mechanisms by which clocks buffer circadian-regulated processes against variations in these environmental time cues (Ruoff et al., 2007; Domijan and Rand, 2011). In plants, quantifying how circadian homeostasis is achieved despite the significant effect of temperature on many biological rate constants (the temperature compensation effect) is an increasingly active research area, with critical implications for crop viability under climate change (Resco et al., 2009).

It has been suggested that the observed complexity in clock transcriptional feedback systems across rhythmic life is essential to buffer timing against noisy environments (Merrow et al., 2005). All rhythmic organisms employ gene regulatory networks to drive circadian output rhythms and all these gene networks share the presence of multiple light inputs and multiple feedback loops, despite the fact that the genes involved are phylogenetically unrelated across higher taxa. These observations suggest that network feedback structure, rather than the precise identity of network components, might be the prominent factor in negating extrinsic noise. The feedback loop structure of clock transcriptional networks has been shown to provide efficient buffering against noisy environmental variables. For example, the circadian clock network of the model fungal species *Neurospora crassa* is built around a central negative feedback loop augmented by an interlocking positive loop (Baker et al., 2012). It has been shown experimentally that the positive loop reduces the variability in the free-running period of the clock (Cheng et al., 2001; Smolen et al., 2001), thereby promoting its robust synchronization to the external LD cycle. Subsequent ODE modeling of the *Neurospora* clock has demonstrated that this interlocked feedback structure also imparts the flexibility necessary to tune the dependence of oscillator phase on both day length (Akman et al., 2008) and ambient temperature (Akman et al., 2010b), yielding a potentially generic mechanism by which temperature compensation can be achieved in a clock network (Akman et al., 2010b). Furthermore, *in silico* evolution studies have suggested that the complex structures emerging from multiple feedback loops and light inputs are an essential property of clock transcriptional networks that enable them to function when subjected to noisy light patterns (Troein et al., 2009). Using a genetic algorithm to evolve a population of random networks into networks that optimally predicted the timing of dawn and dusk, the authors showed that extrinsic noise (daily environmental noise as well as seasonal changes to the photoperiod) is the strongest driver in the selection of complex network structures similar to those observed in nature. These observations, combined with the lack of sequence homology between the actual components of the clock networks, could suggest that the evolving prototype clock systems in the last common ancestor have diverged across taxa by incorporating pre-existing complex structures most suitable for robust timekeeping mechanisms.

*Ostreococcus* could be considered a snapshot of plant evolution around 1.5 billion years ago, before multi-cellularity and terrestrial plants, but after symbiosis with cyanobacteria gave rise to the evolution of the green lineage. Although deep sea ecotypes of *Ostreococcus* exist (Schaum et al., 2013), the sequenced strain of *Ostreococcus* (oth95) used for most laboratory experiments was isolated at the surface of the Thau lagoon in France (Courties et al., 1994). This shallow habitat plus clear waters means that the alga is exposed to extrinsic noise in light intensity at a similar level to that acting on land plants. Temperature fluctuations due to weather are limited, given the substantial body of water. However, there is significant variation in seasonal temperature, as the surface temperature of the lagoon fluctuates between 4 and 29°C (Collos et al., 2009). *Ostreococcus* strain oth95 is thus a suitable model organism to study extrinsic noise in both light and temperature, and it would be interesting to compare the extent of these extrinsic noise effects on the circadian clocks of strains isolated from a range of depths. From limited genetic resources (Corellou et al., 2009), both the experimental system and the mathematical model still exhibit remarkably flexible and robust oscillations when exposed to external noisy light conditions (Troein et al., 2011). However, modeling predicts that this flexibility strictly relies on no less than five independent light inputs, suggesting that in a clock consisting of a few components linked in a simple circuit, a degree of flexibility can be achieved by the added complexity of multiple light inputs. This hypothesis that flexibility can be obtained from complexity in both feedback loop structure and light inputs is further supported by a recent study in which network flexibility was computed using a precise mathematical measure for a range of clock models possessing different network architectures (Dixon et al., 2014). Despite our earlier considerations, it is tempting to speculate that the external noise encountered in a terrestrial multicellular organism would require additional buffering against noise, beyond what can be achieved with multiple light inputs, and has therefore driven the incorporation of additional feedback into the transcriptional clock system. Sustained modeling efforts will be key to systematically analyzing the ability of different network configurations to buffer against external noise.

## CONCLUSION

The studies reviewed here clearly show how introducing intrinsic or extrinsic noise into mathematical models of the plant circadian clock can lead to increased support for experimental studies via the systems biology cycle of iterative model construction/refinement and experimental validation.

An important next step in this field would be to introduce both intrinsic and extrinsic noise into a single computational model of a cellular circadian network to study the interaction of these two types of noise and the effects of noise from a structural as well as functional point of view: i.e., both on clock size and architecture and on precision and robustness.

Another interesting line of work would be the integration of other sources of variability (**Figure 2**), such as humidity changes, and heat and cold stress. Moreover, most circadian models developed to date have focused on the central oscillator network. The introduction of post-translational modifications and the interaction of the clock network with signal transduction and metabolic pathways are also important (Zhang and Kay, 2010; Haydon et al., 2013).

Possibly the most exciting future direction is the development of single-cell mathematical models. Recent advances in experimental techniques now permit single-cell measurements, making it possible to develop, and individually parameterize, models at this scale. Single-cell work has already been carried out in cyanobacteria (Locke and Elowitz, 2009; Yang et al., 2010), and once the technology is available for plants, quantitative single-cell models of the *Arabidopsis* circadian clock could be developed. These could help to establish, for instance, whether coupling contributes to the organ specific properties of the *Arabidopsis* clock (James et al., 2008). Single-cell models would require significant computational resources, as the parameter fitting would have to be applied to a large ensemble of noisy time series datasets, thus making their construction an interesting avenue of research from a computational perspective also.

## AUTHOR CONTRIBUTIONS

All authors contributed equally to this work, read and approved the final manuscript.

## Conflict of Interest Statement

The Associate Editor Dr Naomi Nakayama declares that, despite being affiliated to the same institution as author Dr Gerben van Ooijen, the review process was handled objectively. The authors declare that the research was conducted in the absence of any commercial or financial relationships that could be construed as a potential conflict of interest.
